# Shape and phase control of CdS nanocrystals using cationic surfactant in noninjection synthesis

**DOI:** 10.1186/1556-276X-6-374

**Published:** 2011-05-06

**Authors:** Yu Zou, Dongsheng Li, Deren Yang

**Affiliations:** 1 State Key Laboratory of Silicon Materials and Department of Materials Science and Engineering, Zhejiang University, Hangzhou 310027, People's Republic of China

## Abstract

Monodispersed CdS nanocrystals with controllable shape and phase have been successfully synthesized in this study by adding cationic surfactant in noninjection synthesis system. With the increase of the amount of cetyltrimethylammonium chloride (CTAC) added, the shape of the CdS nanocrystals changed from spherical to multi-armed, and the phase changed from zinc-blende to wurtzite. It was found that halide ion Cl^- ^plays a key role in the transformation, and other halide ions such as Br^- ^can also induce similar transformation. We proposed that the strong binding between Cd^2+ ^and halide ions reduced the reactivity of the precursors, decreased the nuclei formed in the nucleation stage, and led to the high concentration of precursor in the growth stage, resulting in the increase of size and phase transformation of CdS nanocrystals. In addition, it was found that the multi-armed CdS nanocrystals lost quantum confinement effect because of the increase of the size with the increase of the concentration of CTAC.

## Introduction

Colloidal semiconductor nanocrystals have received considerable attention because of their size-dependent properties and applications in fields such as optoelectronic devices [1-3] and biological fluorescence labeling [4,5]. Among those colloidal semiconductor nanocrystals, CdS nanocrystals with controllable size have been successfully synthesized in noncoordinating solvents [6-8], and their physical properties have been extensively investigated. Apart from the size control of the nanocrystals, more and more attention has been devoted to the control of the shape and phase of the nanocrystals [9-15], which can also influence the physical properties of the nanocrystals [16]. Based on the successful synthesis of CdS nanocrystals with various morphologies, the nanocrystals have been introduced into nanocrystal photovoltaic devices [17,18].

For nanocrystal photovoltaic application, semiconductor nanorods or branched nanocrystals are preferable to quantum dots because they naturally provide direct electron transport path which can enhance charge collection efficiency [2]. Peng and co-workers [9,10] have successfully developed a high temperature organometallic method to synthesize CdS nanorods, which has been expanded to synthesize CdS nanowires [11-13]. In this method, alkylphosphonic acid is used as a ligand. Hyeon and colleagues [14] proposed an alternative method to synthesize rod-shaped CdS nanocrystals based on injection of S-oleylamine into hot Cd-oleylamine precursors. Yong et al. [15] investigated the synthesis of rod-shaped CdS nanocrystals in oleylamine systematically and obtained CdS nanocrystals of different shapes by adding different surfactants. Although great advancement has been made in the synthesis of rod-shaped CdS nanocrystals, there still exist some drawbacks in the current methods. First, the chemicals used, such as alkylphosphonic acid and oleylamine, are quite expensive. Second, the current methods are based on hot injection which limits the yield of CdS nanocrystals. Low-cost synthesis of rod-shaped CdS nanocrystals for photovoltaic application in high yield is still a challenge.

Recently, we have proposed a noninjection method to synthesize high-quality size-controllable CdS quantum dots in noncoordinating solvents without nucleation initiators [19]. All the source chemicals used are air-stable and inexpensive. Moreover, hot injection is avoided in this method, and the size of CdS nanocrystals can be easily tuned by changing the concentration of oleic acid (OA) which serves as a ligand. However, the CdS nanocrystals obtained have cubic zinc-blende structure which is highly isotropic, while rod-shaped CdS nanocrystals always have hexagonal wurtzite structure. Therefore, improvements are needed to synthesize rod-shaped CdS nanocrystals using this noninjection method.

In this article, we propose the shape and phase control of CdS nanocrystals based on a noninjection method. We found that addition of cationic surfactant cetyltrimethylammonium chloride (CTAC) could induce the phase transformation of the CdS nanocrystals from cubic to hexagonal structure, resulting in the formation of multi-armed CdS nanocrystals. It was found that halide ion Cl^- ^plays a key role in the transformation, and other halide ions such as Br^- ^can also induce similar transformation. Compared with alkylphosphonic acid and oleylamine, CTAC is quite economic. Therefore, we believe that this approach is suitable for large-scale production of branched CdS nanocrystals for photovoltaic application.

## Experimental section

### Chemicals

Cadmium oxide (CdO, 99.5%) and sulfur (99.5%) were purchased from Shanghai Chemical Reagent Ltd.(Shanghai China). OA (tech. 90%), 1-octadecene (ODE, tech. 90%), and cetyltrimethylammonium hydroxide (CTAOH) were purchased from Aldrich (St. Louis, MO, USA). CTAC and cetyltrimethylammonium bromide (CTAB) were purchased from Aladdin Chemistry (Shanghai China). All the chemicals were used as received without any further purification.

### Synthesis

A mixture of CdO (1 mmol), OA (6 mmol), and 15 mL ODE was degassed at room temperature for 15 min in a three-necked flask and then heated to 280°C under Ar flow. After the CdO was totally dissolved and the solution turned clear to form a Cd-oleate precursor solution, the temperature of the solution was lowered to about 30°C. 0.5 mmol S and cationic surfactant (CTAC, CTAB, or CTAOH) with different amounts were added into the flask. Then, the mixture was heated to 240°C at a rate of approx. 20°C/min and reacted at this temperature for 60 min under a flow of argon gas. The resultant nanocrystals were collected by precipitation with excess ethanol followed by centrifugation, and the product could be redispersed in hexane for characterization.

### Characterization

Transmission electron microscopy (TEM) images were collected using a Philips CM200 transmission electron microscope operating at 160 kV. High-resolution TEM (HRTEM) measurements were carried out using a JEOL 2010F equipment operating at 200 kV. A sample for TEM analysis was prepared by drying a drop of nanocrystal hexane solution on a carbon-coated copper grid and letting it dry in air. X-ray powder diffraction (XRD) was conducted on an X'Pert pro X-ray diffractometer. The samples were prepared by drop casting the nanocrystal hexane solution on glass slides. Ultraviolet-visible (UV-Vis) absorption spectra were recorded on a U-4100 Spectrophotometer.

## Results and discussion

**Figure 1:**
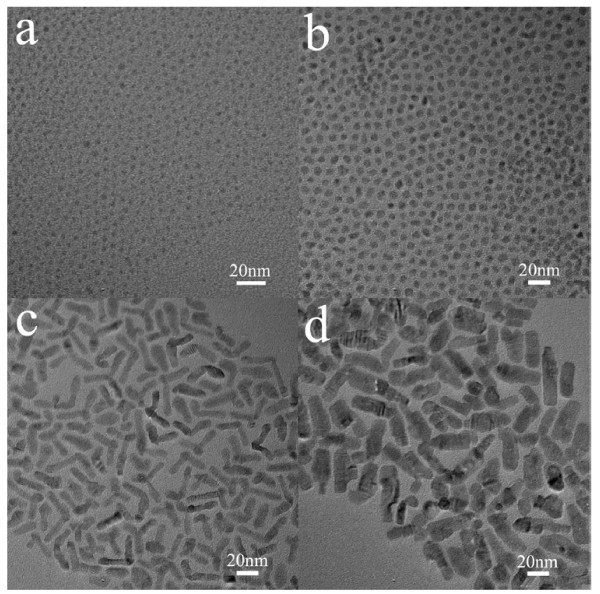
**TEM images of the CdS nanocrystals obtained with different amounts of CTAC added**: **(a) **0 mmol, **(b) **0.25 mmol, **(c) **0.5 mmol, and **(d) **1 mmol. A shape transformation from spherical to multi-armed was observed with the increase of the amount of CTAC.

Selecting proper surfactants has been found to be the key to control the shape and phase of colloidal nanocrystals [20,21]. In our experiments, we chose cationic surfactant CTAC as an additive in the noninjection synthesis of CdS nanocrystals. Figure 1 shows the TEM images of the CdS nanocrystals obtained by adding different amounts of CTAC. As can be seen, the spherical CdS nanocrystals with an average diameter of 3.6 nm were obtained when CTAC was absent. With the increase of the amount of CTAC added, the size of the nanocrystals increased, and the morphology varied from spherical to multi-armed. When 0.5 mmol CTAC was used, a mixture of CdS nanorods, bipods, tripods, and tetrapods with an average diameter of about 7 nm was obtained, which is similar to the products synthesized in oleylamine [14,15]. The average diameter of the multi-armed nanorods further increased to 13 nm when 1 mmol CTAC was added.

**Figure 2:**
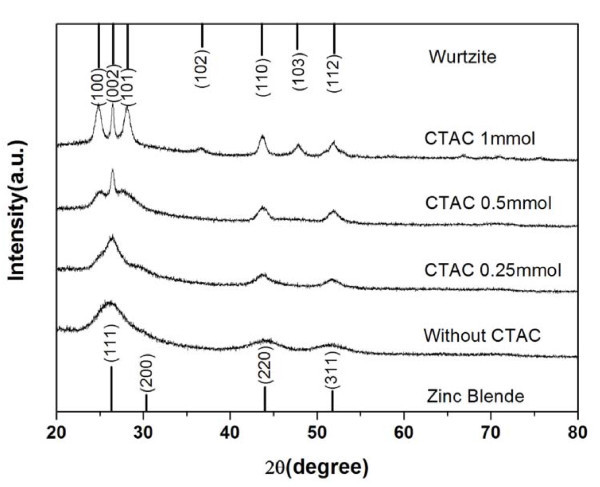
**XRD patterns of the CdS nanocrystals obtained with different amounts of CTAC added**. A phase transformation from zinc blende to wurtzite was exhibited with the increase of the amount of CTAC.

To investigate the morphology transformation induced by the addition of CTAC, XRD analysis was employed for determining the crystal structure of the CdS nanocrystals. The XRD patterns shown in Figure 2 indicate that the phase transformation from zinc blende to wurtzite occurred as the shape of the CdS nanocrystals varied from spherical to multi-armed. When no CTAC was used, the CdS nanocrystals with pure zinc-blende structure were synthesized, which has also been shown in the previous study [19]. With the increase of the amount of CTAC added, the characteristic diffraction peaks of (100), (101), (102), and (103) planes corresponding to the hexagonal wurtzite structure became more and more distinct, revealing the phase transformation from zinc-blende to wurtzite. Meanwhile, the narrowing of the diffraction peaks also indicates the increase of the size of the CdS nanocrystals according to the Scherrer formula, which is consistent with the results obtained from the TEM images.

**Figure 3:**
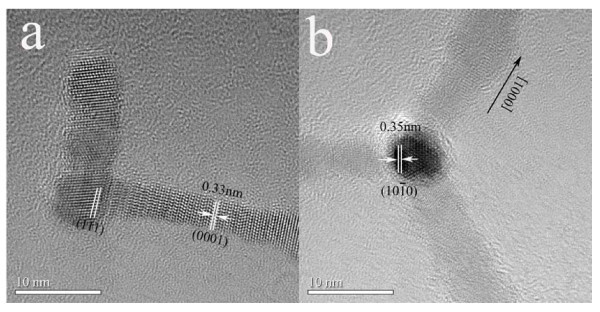
**HRTEM images of the multi-armed CdS nanocrystals synthesized with 0.5 mmol CTAC added**: **(a) **HRTEM image of a typical CdS bipod, consisting of a zinc-blende core and two wurtzite arms; **(b) **HRTEM image of a CdS tetrapod viewed along the [111] direction of the zinc-blende core.

For a more detailed structural characterization of the multi-armed CdS nanocrystals, HRTEM images of the sample with 0.5 mmol CTAC added were recorded. Figure 3a shows a typical HRTEM image of a single CdS bipod. It is obvious that the CdS bipod consists of a zinc-blende core with two wurtzite arms grown out of the (111) facets of the zinc-blende core. The wurtzite arms grow along the [0001] direction which is also confirmed by the cross-sectional image shown in Figure 3b. Figure 3b shows the typical HRTEM image of a CdS tetrapod which is viewed along the [111] direction of the zinc-blende core. It is seen that the cross-sectional plane of the wurtzite arm is that of (0001) which fits well with the (111) plane of the zinc-blende core. From these observations, the crystal nature of the multi-armed CdS nanocrystals is the same as that of other nanotetrapods reported in the literature [22-26]. The formation of the CdS multi-armed nanocrystals is the result of nucleation of zinc-blende cores followed by surface-initiated growth of wurtzite arms. The low yield of tetrapods in the product mixture is probably attributed to the low precursor concentration in the reaction mixture [14,15].

**Figure 4:**
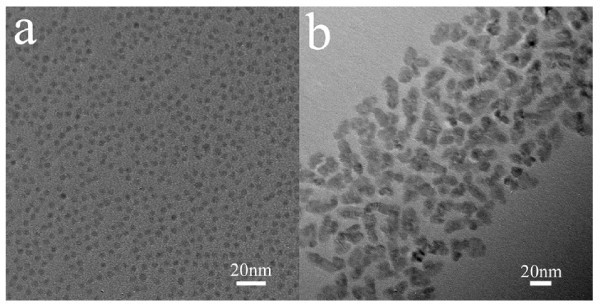
**TEM images of the CdS nanocrystals synthesized with the addition of different cationic surfactants**: (a) 0.5 mmol CTAOH and (b) 0.5 mmol CTAB.

**Figure 5:**
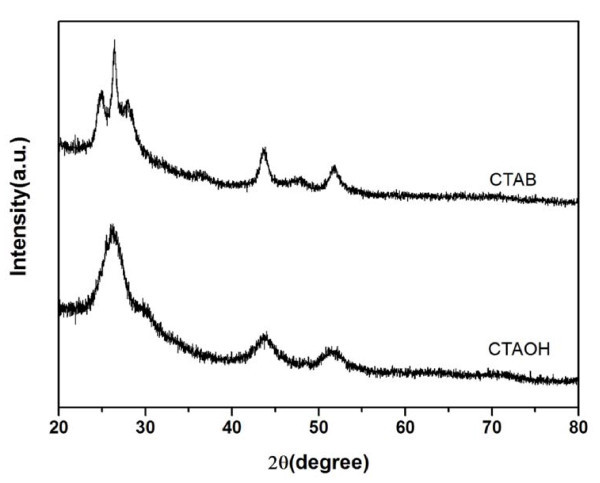
**XRD patterns of the CdS nanocrystals synthesized with 0.5 mmol CTAOH or 0.5 mmol CTAB added**. CTAB can induce the phase transformation of CdS nanocrystals while CTAOH cannot.

Compared to our previous study [19], the only difference in this study is the addition of CTAC into the reaction mixture, and so CTAC must be the key to control the shape and phase of the CdS nanocrystals. Although it has been found that cationic surfactant can induce the formation of CdSe tetrapods, the exact role that cationic surfactant played in the reaction is still not clear. Wong and colleagues [24] suggested the degradation product of cationic surfactant at high temperature might be responsible for the phase transformation. To gain insight into the role of cationic surfactant, we replaced CTAC with other cationic surfactants. When CTAOH was used, amines should be the degradation products which are well known as activation agents for nanocrystal growth and which can induce the formation of wurtzite structure [27]. However, from the TEM image of the CdS nanocrystals synthesized with 0.5 mmol CTAOH shown in Figure 4a, we found that the size and shape changed little although the mean diameter of the CdS nanocrystals increased to 4 nm because of the existence of amines. While CTAB was used, remarkable morphology transformation was observed again. XRD analysis (Figure 5) further confirms the phase transformation that occurred when CTAB was used, while the nanocrystals still had zinc-blende structure when CTAOH was used. Therefore, we believe that halide ions play a key role in the shape and phase control of CdS nanocrystals.

Recently, other research groups also found that halide ions could influence the growth of CdS nanocrystals significantly. Saruyama et al. [28] reported zinc-blende CdS seed nanoparticles could be transformed to wurtzite nanorods through an Ostwald ripening process induced by Cl^- ^and the surfactants OA and oleylamine. Tai et al. [29] showed that wurtzite CdS nanoparticles could be synthesized by the addition of NaCl in an ultrasound-assisted microwave synthesis system. Therefore, it is believed that the presence of Cl^- ^favors the formation of wurtzite CdS. According to the "hard-soft acid-base model," [30] Cd^2+ ^is a soft acid, and Cl^- ^is a soft base. Soft acids bind strongly to soft base, so Cl^- ^binds strongly to Cd^2+^, which reduces the reactivity of the precursors. As a result, the nuclei formed in the nucleation stage decrease, and the precursors remained for the growth of the nuclei increase, leading to the formation of larger CdS nanocrystals. Earlier study has proven that the structure of CdS nanocrystals is size dependent [31-33]. The zinc-blende structure is dominant at small diameter (approx less than 4 nm), and wurtzite structure is more important at large diameters (approx greater than 5 nm). Therefore, the phase transformation from zinc-blende to wurtzite happens with the addition of CTAC or CTAB. Moreover, at high precursor concentration, relative difference between the growth rates of different faces can lead to rod-shape nanocrystals [34]. However, in contrast with above mentioned study, we obtained nanocrystals with more complex structure consisting of zinc-blende cores and wurtzite arms. The formation of zinc-blende cores can be attributed to the magic-sized nuclei formed at the nucleation stage. It has been found that these nuclei all have zinc-blende structure which is more suited than the corresponding wurtzite structure to the formation of closed-shell formations and thus are more stable [10]. Unfortunately, we failed to separate these nuclei because of the high reaction rate. It was observed that multi-armed CdS nanocrystals had been formed when the reaction temperature reached 200°C. When the nanocrystals is more than 4 nm in diameter, the growth of wurtzite phase is more favored. At high precursor concentration, the faster growth rate of (0001) plane leads to the growth of wurtzite arms. As a result, the multi-armed CdS nanocrystals with zinc-blende cores and wurtzite arms are formed.

**Figure 6:**
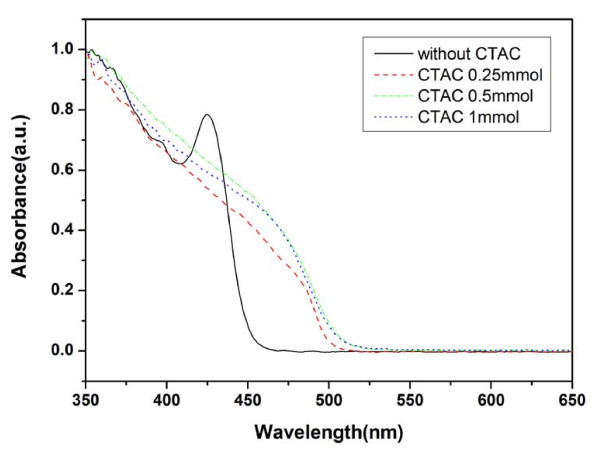
**UV-Vis absorption spectra of the CdS nanocrystals obtained with different amounts of CTAC added**. The multi-armed nanocrystals lose quantum confinement effect.

Figure 6 depicts the UV-Vis absorption spectra of the CdS nanocrystals prepared with different amounts of CTAC added. For the spherical CdS nanocrystals synthesized without CTAC, a strong exciton peak at 425 nm appears because of quantum confinement effect. The narrow peak also reveals narrow size distribution. With the increase of the amount of CTAC added, the absorption peak red-shifts and gradually disappears due to the increase of the size of CdS nanocrystals. Since the size of the multi-armed CdS nanocrystals is far beyond the exciton Bohr radius of CdS (approx. 3 nm), they lose quantum confinement effect, confirmed by the absorption onset located at approx. 512 nm, matching well with the band gap energy of bulk CdS (2.42 eV).

## Conclusions

We report the successful shape and phase control of CdS nanocrystals using cationic surfactant CTAC in noninjection synthesis. Multi-armed CdS nanocrystals with zinc-blende cores and wurtzite arms are synthesized economically, which are suitable for nanocrystal photovoltaic application. It is confirmed that Cl^- ^must be responsible for the phase transformation from zinc blende to wurtzite, and other halide ions such as Br^- ^can also induce similar transformation. It is believed that this scheme can be extended to synthesize other semiconductor nanocrystals with complex structure.

## Abbreviations

CTAB: cetyltrimethylammonium bromide; CTAC: cetyltrimethylammonium chloride; CTAOH: cetyltrimethylammonium hydroxide; HRTEM: high-resolution TEM; OA: oleic acid; ODE: 1-octadecene; TEM: transmission electron microscopy; UV-Vis: ultraviolet-visible; XRD: X-ray powder diffraction.

## Competing interests

The authors declare that they have no competing interests.

## Authors' contributions

ZY carried out the experiments and drafted the manuscript. LD participated in the discussion of the results and in revising the manuscript. YD supervised the work and revised the manuscript. All authors read and approved the final version of the manuscript.
